# Progressive Telomere Dysfunction Causes Cytokinesis Failure and Leads to the Accumulation of Polyploid Cells

**DOI:** 10.1371/journal.pgen.1002679

**Published:** 2012-04-26

**Authors:** Judit Pampalona, Cristina Frías, Anna Genescà, Laura Tusell

**Affiliations:** Department of Cell Biology, Physiology, and Immunology, Bioscience School, Universitat Autònoma de Barcelona, Bellaterra, Spain; University of Washington, United States of America

## Abstract

Most cancer cells accumulate genomic abnormalities at a remarkably rapid rate, as they are unable to maintain their chromosome structure and number. Excessively short telomeres, a known source of chromosome instability, are observed in early human-cancer lesions. Besides telomere dysfunction, it has been suggested that a transient phase of polyploidization, in most cases tetraploidization, has a causative role in cancer. Proliferation of tetraploids can gradually generate subtetraploid lineages of unstable cells that might fire the carcinogenic process by promoting further aneuploidy and genomic instability. Given the significance of telomere dysfunction and tetraploidy in the early stages of carcinogenesis, we investigated whether there is a connection between these two important promoters of chromosomal instability. We report that human mammary epithelial cells exhibiting progressive telomere dysfunction, in a pRb deficient and wild-type p53 background, fail to complete the cytoplasmatic cell division due to the persistence of chromatin bridges in the midzone. Flow cytometry together with fluorescence *in situ* hybridization demonstrated an accumulation of binucleated polyploid cells upon serial passaging cells. Restoration of telomere function through hTERT transduction, which lessens the formation of anaphase bridges by recapping the chromosome ends, rescued the polyploid phenotype. Live-cell imaging revealed that these polyploid cells emerged after abortive cytokinesis due to the persistence of anaphase bridges with large intervening chromatin in the cleavage plane. In agreement with a primary role of anaphase bridge intermediates in the polyploidization process, treatment of HMEC-hTERT cells with bleomycin, which produces chromatin bridges through illegimitate repair, resulted in tetraploid binucleated cells. Taken together, we demonstrate that human epithelial cells exhibiting physiological telomere dysfunction engender tetraploid cells through interference of anaphase bridges with the completion of cytokinesis. These observations shed light on the mechanisms operating during the initial stages of human carcinogenesis, as they provide a link between progressive telomere dysfunction and tetraploidy.

## Author Summary

Chromosome instability leads to the accumulation of chromosome number and structure aberrations that have been suggested as necessary for neoplastic transformation. Telomeres, specialized DNA–protein complexes localized at the physical ends of linear chromosomes, are crucial for maintaining chromosome stability. Massive chromosomal instability may occur when cells continuously proliferate in the absence of specific telomere elongation mechanisms. Besides telomere dysfunction, it has been suggested that a transient phase of tetraploidization has a causative role in cancer. This study provides a link between dysfunctional telomeres and the generation of tetraploids. Using a human mammary epithelial cell model, we show that diploid cells exhibiting progressive telomere dysfunction, in a p53 proficient background, engender tetraploid cells through cytokinesis failure. Our studies give new insights into the mechanisms that may facilitate the evolution of malignant phenotypes: telomere-dependent chromosome instability would engender tetraploid intermediates that, on division, would promote further cellular genome remodelling, which is needed at the early stages of tumour development in order for cells to become neoplasic.

## Introduction

Most cancer cells are genetically unstable [1] and accumulate unbalanced chromosome rearrangements, entire chromosome aneuploidies and increased numbers of chromosome sets (Mitelman Database: http://cgap.nci.nih.gov/Chromosomes/Mitelman). Cell populations with chromosome contents from 42 to 95 are often found in prostate, pancreas, ovary, large intestine, liver and breast adenocarcinomas, as well as in squamous cell carcinomas of the skin [2]. Moreover, tetraploidy is usually observed in the early stages of cervical carcinogenesis [3] and in a pre-malignant condition called Barrett's esophagus, in which tetraploid cells have been correlated with the loss of p53 and detected before gross aneuploidy occurs [4], [5].

A long-standing hypothesis on tumourigenesis suggests that unstable tetraploid cells (4N) can act as intermediates that catalyze the generation of aneuploid cells [6]–[8]. This assumption is based on several studies that show that tetraploidy leads to increased chromosome instability in eukaryotic cells [9]–[11]. When cells become tetraploid, they acquire extra centrosomes that can potentially lead to chaotic multipolar mitosis, in which sister chromatids are frequently missegregated between daughter cells (reviewed by [12], [13]). Tumourigenesis via this tetraploid intermediate could explain why polyploid cells are observed in early neoplastic stages, and why cancer cells frequently contain supernumerary centrosomes and a high rate of whole chromosome missegregation. The most direct evidence for the high tumourigenic potential of tetraploid cells comes from the observation that 4N p53-null mammary epithelial mouse cells can initiate tumours in immunocompromised mice, whereas isogenic diploids cannot [10]. This evidence supports the idea that tetraploidy is an intermediate for chromosome instability (CIN) and tumourigenesis.

Besides bearing near-tetraploid genomes, another feature of human tumour cells is the presence of very short telomeres [14]. Telomeres are nucleoprotein complexes that form a loop structure at the end of chromosomes protecting them from end-to-end fusion. Excessive telomere shortening due to continuous cell proliferation in an environment with checkpoint deficiencies promotes the appearance of uncapped chromosome ends that may initiate repeated breakage-fusion-bridge cycles. This leads to massive CIN visualized as complex types of genomic abnormalities, including loss of heterozigosity, gene amplification, chromosome reorganizations and whole-chromosome missegregation, which are characteristic of most human tumour cells. It is currently believed that telomere-attrition-induced genomic instability contributes to the onset of epithelial carcinogenesis. Indeed, short dysfunctional telomeres in mouse models proved to have a founder effect in CIN and tumourigenesis [15]. In humans, telomere dysfunction occurs during normal ageing, and critically short telomeres have been reported as a common early alteration in many epithelial cancers [16], [17], the prevalent tumour type in the elderly.

Given the coexistence of both telomere dysfunction and tetraploidy in early human-cancer lesions, we investigated the possible connection between these two important promoters of CIN. For this purpose, we used human epithelial cells (HMECs) derived from normal mammary gland. In these cells, spontaneous *P16INK4A* promoter hypermethylation overrides the pRB pathway and allows cells with critically short telomeres to proliferate [18]. When this occurs, uncapped chromosome ends may gradually be repaired in the form of end-to-end chromosome fusions. These chromosome configurations tend to bridge at anaphase if a twist in their intercentromeric region occurs. We have already shown that these chromatin bridges can break and give rise to structural chromosome rearrangements and amplification events [19], [20], or alternatively, a small portion of unbroken chromatin bridges can segregate erroneously during mitosis between daughter HMECs, causing whole-chromosome aneuploidy [21]. In this current article, we demonstrate that progressive telomere attrition, in a context of functional p53, can also result in the generation of polyploid binucleated cells when long-lasting unbroken DNA bridges span the cleavage site and interfere with the completion of cytokinesis.

## Results

### Telomere dysfunction causes the accumulation of extra chromosome sets in primary HMECs

**Figure 1:**
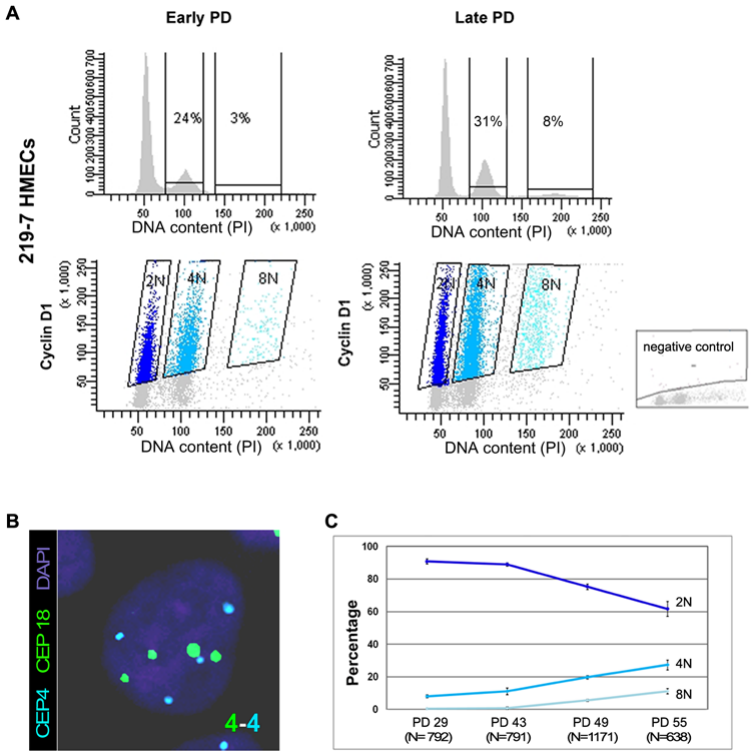
Replicative dependent polyploidization in HMECs. (A) Flow cytometry analysis of DNA content of 219-7 HMECs stained with propidium iodide, at early and late culture PDs (upper panels). The percentage of cells with 4N and 8N DNA content is given. Bivariate analysis of DNA content and expression of cyclin D1 protein (lower panels), in which cyclin D1 positive cells are grouped depending on their ploidy level. Negative control, shown in the bottom right corner, was performed without the primary antibody. (B) FISH analysis of a polyploid cell nucleus showing four centromere signals corresponding to chromosome 4 (in blue) and four signals corresponding to chromosome18 (in green). DNA is counterstained with DAPI. (C) Graph illustrating ploidy evolution of 219-7 HMECs throughout the culture based on a cell per cell basis scoring of the number of centromeric signals. Average percentages from two independent experiments with standard deviations are shown.

HMECs cultured *in vitro* in a serum-free medium exhibit eroding telomeric sequences and ultimately enter a telomere-based crisis, generating the types of chromosomal abnormalities seen in the earliest lesions of breast cancer. Early studies using the HMEC model revealed the presence of structural [18]–[20] and numerical chromosome aberrations [21], as well as certain levels of polyploidy [18]. In order to further investigate the emergence of polyploid subpopulations of HMECs, we examined their DNA content throughout the cell culture by univariate flow cytometry. We focused this analysis on cells derived from breast specimens from two different donors (219-7 and 830), in order to take interindividual variations into account. Figure 1A, upper panels, shows the results of the cytometric analysis for donor 219-7. A marked increase in the 4N fraction was observed in late-passage HMECs compared with their early counterparts (from 24% to 31%). The cytometric analysis of HMECs derived from the second donor also demonstrated a significant accumulation of 4N cells with population doublings (PDs) (Figure S1). To check whether the increase in cells in the 4N fraction actually reflected an increase in tetraploid cells in G1 and not in diploid cells with replicated DNA, we performed a flow-cytometry bivariate analysis of both the DNA content and cyclin D1 protein, which is only expressed during the G1 cell-cycle phase [22]. This approach showed that a portion of cells in the 4N window were positive for cyclin D1 in both donors; they therefore exhibited *bona fide* traits of real tetraploidy (Figure 1A lower panels and Figure S1). In agreement with an increase of extra chromosome sets with cell culture, the cell-cycle analysis also revealed a small fraction of 8N cells that increased in both donors at late PDs. To reinforce these results, we also checked for ploidy levels on a cell-per-cell basis. By scoring centromeric signals of two different chromosomes in donor 219-7 (Figure 1B), we confirmed that there was a polyploid cell subpopulation that increased progressively throughout the culture (Figure 1C). The frequency of tetraploid cells increased from a basal level of 7.97% at an early PD to 27.13% at PD55 (Kruskal Wallis, *post-hoc* Dunnett's test, p<0.001). Collectively, our results show that there is an accumulation of tetraploid cells throughout the HMEC culture. And remarkably, this 4N population is viable and able to proliferate because an octoploid population of cells emerges at the later stages of the culture.

In addition to the increasing frequencies of polyploidy, HMECs also display an increasing grade of telomere dysfunction with continuous proliferation [19], [21]. To ascertain whether there is a correlation between these two parameters, we scored the frequency of chromosome ends with undetectable PNA-FISH telomeric signals and ploidy levels in metaphase spreads from HMECs at different PDs. The lowest levels of telomere dysfunction were observed at the earliest PDs, coincident with the lowest tetraploidy rate in the two donors. At late PDs, telomere dysfunction affected a greater number of chromosome arms (Figure S2A), and tetraploidization events also increased. The Spearman statistical test showed a significant correlation between tetraploidy and telomere dysfunction (r^2^ = 0.895; p<0.05; Figure S2B). These results suggest that the emergence of polyploid cell populations is probably linked to the natural telomere erosion that HMECs undergo as they proliferate.

**Figure 2:**
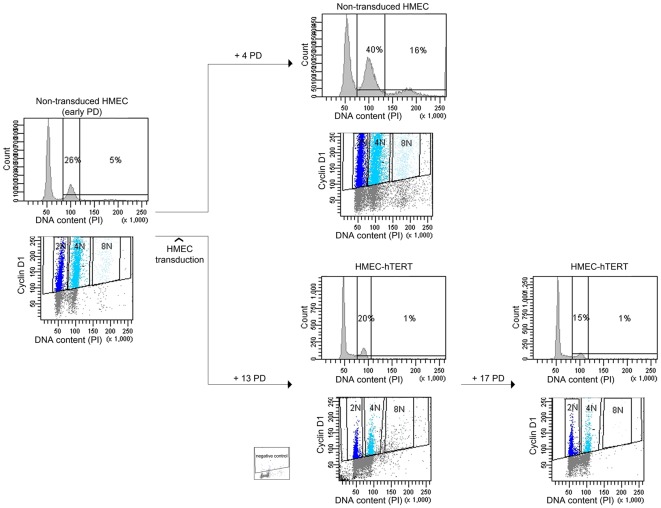
Polyploidization is reverted by hTERT immortalization. DNA content evolution of non-transduced and hTERT-transduced HMECs at different PDs by univariate and bivariate flow cytometry. At an early PD, non-transduced 219-7 HMECs were analyzed for the presence of tetraploidy (left panels). Cells were then divided into two groups. Proliferation of non-transduced HMECs resulted in the accumulation of a 4N subpopulation (upper right panels). In contrast, proliferation of HMEC-hTERT cells revealed a sharp reduction of the 4N subpopulation (lower right panels). Figure shows plot with negative control.

To better establish a relationship between telomere dysfunction and the generation of polyploid cells, we transduced HMECs with the catalytic subunit of telomerase (hTERT), as introduction of hTERT leads to the elongation of telomeric DNA ends and cell immortalization [23]. Finite lifespan HMECs 219-7 at early PDs were exposed to lentiviral particles containing an hTERT construct. Expression of telomerase activity in hTERT-transduced cells was verified by TRAP assay (data not shown) and the restoration of telomere length was assessed by analyzing free-telomere chromosome ends after PNA-FISH (Figure S2C). The evolution of ploidy levels of non-transduced HMECs and hTERT-transduced cells (HMEC-hTERT) was followed by flow cytometry (Figure 2). Whereas extra chromosome sets in non-transduced HMECs increased significantly throughout the cell culture, the reactivation of telomerase was accompanied by a significant decrease in the frequency of polyploid cells (Student's T test; p<0.05; Figure 2). Restoration of telomerase in HMECs from two additional donors also resulted in a notable reduction of the polyploid levels (data not shown). Together, these results evidenced the connection between telomere dysfunction and polyploidization, as telomerase re-introduction rescued the polyploid phenotype of the epithelial cells.

### Polyploid HMEC subpopulations arise due to incomplete cytokinesis

The above results prompted us to focus on how telomere shortening might promote the formation of tetraploid cells. In *Drosophila*, unprotected telomeres trigger the spindle assembly checkpoint that prevents cells from progressing into anaphase [24]. Moreover, deprotection of chromosome ends in TRF2-deficient mouse hepatocytes impedes the onset of anaphase without affecting DNA replication [25]. Similarly, persistent telomere deprotection due to deletion of the telomeric DNA-binding protein POT1a/b in p53-defective mouse embryonic fibroblasts leads to cell tetraploidization [26] through endoreduplication [27]. It has also been suggested that this latter mechanism underlies whole-genome duplications in faulty p53 colon cancer human cell lines where telomerase activity was abrogated [28]. In all these cases, cells skip or exit mitosis without undergoing anaphase or cytokinesis and give rise to tetraploid cells with a single nucleus. Nevertheless, polyploidy can also appear due to cytokinesis failure after karyokinesis has been completed, thus giving rise to polyploid binucleated cells. Specifically, telomere dysfunction could be envisaged as a factor potentially capable of interfering with the completion of cytokinesis through anaphase bridges resulting from end-to-end chromosome fusions. This possibility is based on the observation that the presence of bulk chromatin occluding the cleavage plane is known to induce furrow regression [29]–[31].

**Figure 3:**
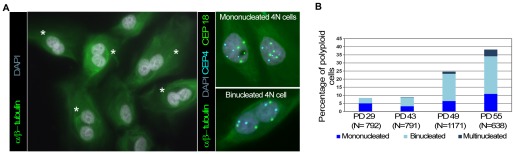
Polyploid HMECs arise through incomplete cytokinesis. (A) Immunofluorescence of alpha and beta-tubulin and DAPI staining allows binucleated (white asterisks) to be distinguished from mononucleated HMECs (left image). ImmunoFISH combining alpha and beta tubulin together with centromeric specific DNA probes for chromosome 4 (in blue) and 18 (in green) revealed two tetraploid cells each with a single 4N nucleus (top right), and a tetraploid cell containing two 2N nuclei within the same cytoplasm (down right). (B) Averaged percentage of mononucleated, binucleated and multinucleated polyploid HMECs throughout the cell culture from two different experiments.

To determine the mechanism by which polyploidy emerges in an environment where progressive telomere dysfunction occurs, we scored for the presence of binucleated and mononucleated cells throughout the HMEC culture. Antibodies against alpha and beta-tubulin allowed the cell boundaries to be delineated and DNA content was determined by dual-color centromeric FISH (Figure 3A). Most tetraploid and octoploid cells contained two nuclei in a single cytoplasm, which is incompatible with an endoreduplication or mitotic slippage origin but consistent with cytokinesis abortion (Figure 3B). However, a fraction of mononucleated polyploid cells was also observed (Figure 3B). These cells did not exhibit signs of endoreduplication cycles, as FISH signals were randomly distributed within the interphase cell nucleus (Figure 3A). In addition, most metaphase spreads of HMECs exhibit two chromatids per chromosome (Figure S2D); only a very low frequency (∼0.75%; 8 out of 1057) of endoreduplicated karyotypes, in which chromosomes were present as diplochromosomes (Figure S2E), were observed. Thus, 4N mononucleated HMECs probably emerged from the division of an existing binucleated polyploid cell, as it is already known that binucleated cells form a single metaphase plate in the following mitosis [32].

**Figure 4:**
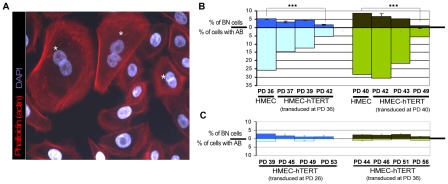
Quantification of anaphase bridges and binucleates in non-transduced and hTERT-transduced HMECs. (A) Texas Red-X phalloidin labelling of actin filaments and DNA counterstaining with DAPI of non-transduced HMECs. White asterisks highlight those binucleated cells. (B) Analysis for the presence of binucleated cells (BN; upper bars) and anaphase bridges (AB; lower bars) in non-transduced HMECs and at different PDs immediately after transducing cells with hTERT. Two independent cell transduction experiments were performed (transduction at PD36, in blue and at PD40, in green). (C) The same analysis was performed after HMEC-hTERT cells were passaged more than 8 PDs after transduction. Again, two independent transduction experiments were performed (transduction at PD26, in blue and at PD36, in green).

To further test the idea that the polyploidization associated with progressive telomere dysfunction is due to cytokinesis failure, we investigated whether restoration of telomerase influenced the spontaneous formation of binucleates. For this purpose, mono- and binucleated cells were scored in non-transduced HMECs and along a short follow up after hTERT transduction. The number of nuclei in each cell was recorded after applying phalloidin-texas red to detect the cell cortex and DAPI staining to counterstain DNA (Figure 4A). The analyses showed a gradual decrease in the frequency of spontaneous binucleated cells after telomerase restoration (Figure 4B upper bars). Of particular noteworthiness, a significant reduction of spontaneous binucleates was observed in HMEC-hTERT cells as early as 6 PDs after transduction, when compared to non-transduced HMECs (Kruskal-Wallis, *post-hoc* Dunnett's test p<0.001). Together, these results demonstrate that restoration of telomerase had a strong effect in reducing the spontaneous formation of binucleated cells and suggest that short dysfunctional telomeres in HMECs may promote the formation of polyploid cells by interfering with the completion of cytokinesis.

### The presence of bridged chromatin *per se*, and not telomere dysfunction, induces cell tetraploidization

Telomere shortening below a critical length results in uncapped chromosomes prone to fuse with each other, which may potentially interfere with proper cytokinesis if they form a bridge during mitosis. If this were the case, increasing levels of telomere-dysfunction would be expected to correlate with higher probabilities of anaphase bridges and cytokinesis failure, whereas elongation of critically short telomeres by telomerase would be expected to attenuate binucleate formation by recapping chromosome ends. In accordance with these assumptions, we observed that restoration of telomerase in HMECs was quickly translated into a sharp decrease of anaphase bridges during the firsts PDs immediately after transduction (Kruskal-Wallis, *post-hoc* Dunnett's test, p<0.001; Figure 4B lower bars), which in turn was paralleled with the reported decrease in the frequency of spontaneous binucleated cells (Figure 4B upper bars). Interestingly, immortalization of HMECs with hTERT did not completely abrogate CIN. Indeed, cells passaged for more than 20PDs, after hTERT transduction, presented a low basal frequency of anaphase bridges that again correlated well with a residual level of binucleates (Figure 4C). Together these results strongly suggest a connection between the presence of anaphase bridges and the emergence of binucleated cells.

**Figure 5:**
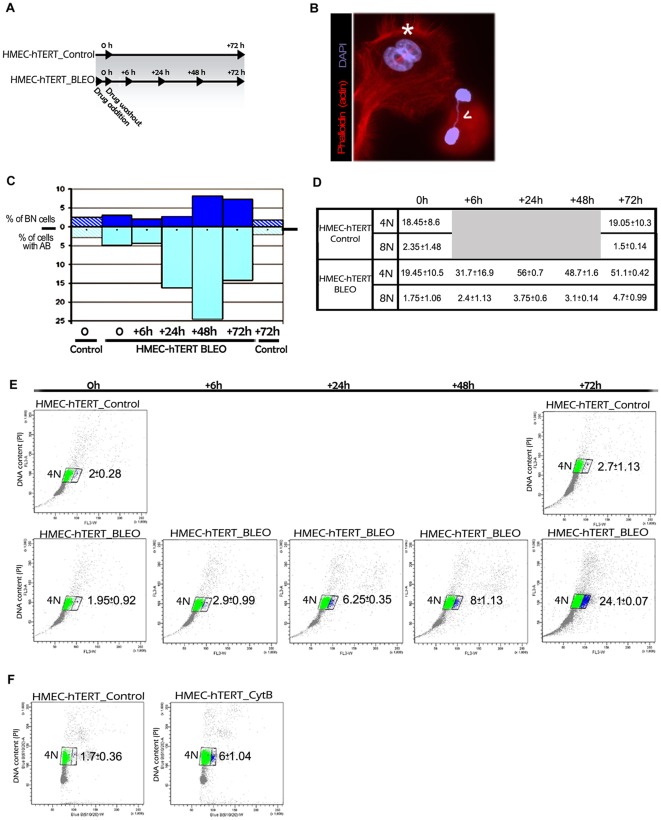
Artificially induced anaphase bridges engender binucleated polyploid HMEC-hTERT cells. (A) Immortalized HMECs were treated for one hour with the DNA damage-inducing agent bleomycin (HMEC-hTERT_BLEO) to induce double strand breaks. After drug washout, cells were collected at different timepoints (6, 24, 48 and 72 h after treatment). Non-treated cells (HMEC-hTERT_Control) were also collected in parallel at 0 and 72 hours. Then DNA content was analyzed by flow cytometry and the presence of binucleated cells and anaphase bridges was scored after Texas Red-X phalloidin and DAPI staining. (B) Image showing a binucleated cell (white asterisk) and two nuclei connected by a chromatin bridge (open white arrow). (C) The graph shows the evolution of the percentage of BN and AB on HMEC-hTERT_BLEO (non-patterned bars) and HMEC-hTERT_Control (stripped bars). (D) The table resumes the DNA content values (averaged from three different experiments) resulting from flow cytometry analyses. Standard deviation is shown. (E) Plots resulting from the analysis of the DNA content (Y axes) and the forward light scatter (FLS) (X axes), which allows for the identification of larger cells. Of note, upon drug treatment, a cell subpopulation with a larger area appears (blue dots). An averaged value from three independent experiments is shown. (F) HMEC-hTERT cells were treated with cytochalasin B (HMEC-hTERT_CytB), during 24 hrs before FACS sorting, to artificially obtain binucleated cells. The dot plot shows that the fraction of cells falling in the right gate (in blue) increases upon Cyt-B treatment. Averaged data and standard deviation from three independent experiments is shown.

To obtain additional insight into the effect of bridged chromatin in the generation of polyploid subpopulations, we treated HMEC-hTERT cells with the DNA-damaging agent bleomycin in order to obtain anaphase bridges unrelated to telomere dysfunction. An appropriate concentration of the drug was added to the culture medium over one hour to induce DNA breaks. Treated cells were analyzed at 0 h, 6 h, 24 h, 48 h and 72 h after drug washout (WO); a control group of untreated cells was also analyzed at 0 h and 72 h (Figure 5A). After DNA damage is inflicted, broken DNA ends can join illegitimately, producing chromosome rearrangements such as dicentric chromosomes that, similarly to end-to-end fusions, may bridge during anaphase. Therefore, an accumulation of dicentric chromosomes is expected to occur in the first mitoses after damage, and chromatin bridges are expected to raise the population of binucleated cells at the next interphase. Accordingly, 24 h after drug release, a significant increase in the number of cells showing anaphase bridges was observed (Kruskal-Wallis, *post-hoc* Dunnett's test, p<0.001; Figure 5B and 5C lower bars). This frequency significantly rose at 48 h post WO and declined after 72 h, in accordance with the preferential breakage fate of chromatin bridges during cell division [33], [34]. But more importantly, a significant rise in the frequency of binucleates was observed at 48 h and 72 h post WO (Kruskal-Wallis, *post-hoc* Dunnett's test, p<0.001; Figure 5B and 5C upper bars), which was consistent with anaphase bridges impeding proper cytokinesis.

In order to better connect the formation of anaphase bridges with the observed increase in ploidy levels, bleomycin treated and untreated HMEC-hTERT cells were monitored for DNA content by univariate cytometric analysis at different time intervals. Treatment of cells with bleomycin resulted in an accumulation of 4N cells over time, which is compatible with aborted cytokinesis (Figure 5D). Strikingly, when analyzing the cells' 4N gate, a proportion of cells with a larger size was observed to increase with time after treatment (Figure 5E). This subpopulation of bigger cells was not observed in any of the control groups (0 h and 72 h), nor in bleomycin-treated cells at initial times post WO. As a whole, these results prompted us to determine whether the gain in cell volume was associated with the presence of binucleated cells. To this end, we treated HMEC-hTERT cells with the mycotoxin cytochalasin B (Cyt-B) to artificially induce binucleated cells, as it inhibits cytoplasmic division by blocking the contractile actomyosin ring. The cytometric analysis of Cyt-B treated HMEC-hTERT cells revealed an accumulation of 4N cells, but even more important, a significant rise in the proportion of enlarged cells was observed (Student's T test; p<0.001; Figure 5F). The microscopic analysis of this fraction of cells after sorting confirmed an enrichment for binucleates in the Cyt-B treated vs the untreated control cells (94% vs 17%). Altogether, these data suggest that DNA bridges, either due to progressive telomere dysfunction or to illegitimate DSB repair, lead to cell polyploidization through the interference of chromatin with the completion of cytokinesis.

### Persistent trapped chromatin causes furrow regression and tetraploidy

Cytokinesis, the final step in cell division, is a highly ordered process divided into four stages, which include specification of the cleavage plane, ingression of the cleavage furrow, formation of the midbody, and abscission. For successful cell division a proper execution of the different coordinated steps is required, and interference with any of these may result in cytokinesis failure and the emergence of tetraploid cells. In order to investigate how bridged chromatin may obstruct cytokinesis, we examined cell divisions of HMECs transiently expressing the histone H2B-GFP fusion protein by time-lapse microscopy. We followed 56 H2B-GFP-HMECs with and without chromatin bridges during a maximum recording period of 3 h. The average time required to complete furrow ingression after anaphase onset in cells without bridges (n = 28) was found to be 13.1±2.3 min. This time period was not significantly different from that needed by cells displaying chromatin bridges (Mann-Whitney, p = 0.9670). Therefore, the presence of bridged chromatin in cells did not appear to interfere with either cleavage plane specification or cleavage furrow ingression.

**Figure 6:**
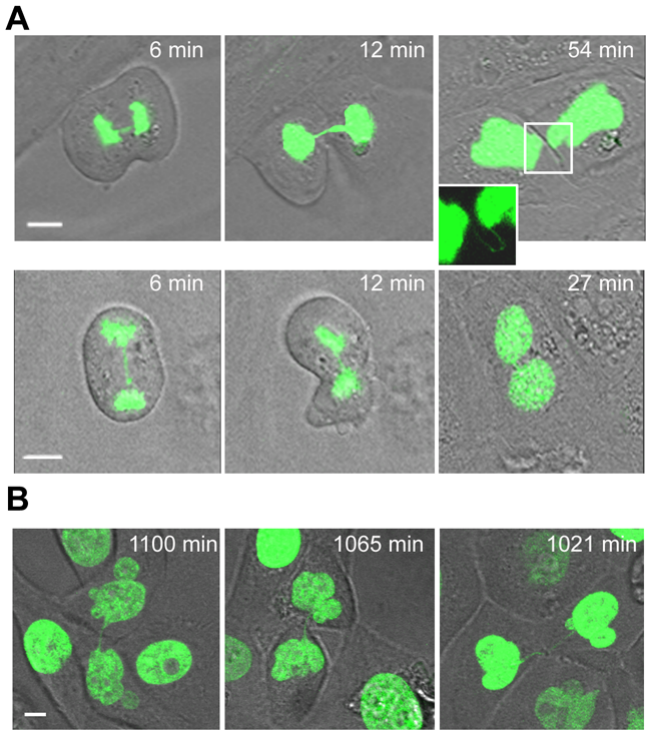
Unbroken anaphase bridges interfere with abscission in HMECs and HMEC-hTERT cells. Non-transduced and hTERT-transduced HMECs were transfected with a H2B-GPF plasmid. (A) Time-lapse micrographies of non-transduced HMECs transiently expressing H2B-GFP. Time 0 was set as the last time point before anaphase onset. Upper images show progressively strengthened unbroken anaphase bridges that remain undisrupted connecting the two nuclei (see inset). Lower images show the formation of a binucleated cell where this process is coupled to the presence of an unbroken chromatin bridge. The scale bar represents 10 µm. (B) Three examples of HMEC-hTERT_BLEO expressing H2B-GFP with over strengthened chromatin bridges long time after anaphase onset. Scale bar represents 10 µm.

Live cell imaging also provided information on the fate of cells with anaphase bridges. In most cells (18 out of 28) bridge breakage occurred and cells eventually completed division. Cells with unbroken bridges had two different outcomes: 70% (7 of 10 cells with unbroken bridge/s) did not complete abscission but resulted in two daughter cells connected with an ultra-fine DNA string that persisted during the whole recording period (Figure 6A upper panels). The remaining 30% (3 of 10 cells with unbroken bridge/s) resulted in a binucleated cell at the end of mitosis (Figure 6A lower panels), thus clearly evidencing that polyploidization is mediated by unbroken chromatin bridges interfering with cytokinesis completion.

The rapid emergence of binucleated cells (at 34.3±8.1 min after anaphase onset, not different from the 30.4±4 min needed by normal segregating cells to become completely attached to the surface; Mann-Whitney, p = 0.421), was quite unexpected because it has been reported that DNA fibers occluding the cleavage plane delay abscission to prevent tetraploidization [35]. Therefore, with the aim of elucidating whether nuclear strings that persist for more than 3 h may give rise to binucleated cells later on in G1, we again tracked HMECs but for a longer period (22 h). This long-term live-cell imaging was performed using bleomycin-treated H2B-GFP-HMEC-hTERT cells (n = 14), in order to increase the formation of anaphase bridges. Again, cells showing chromatin bridges exhibited different fates: completed division after bridge breakage (n = 5), originated binucleated cells (n = 3) or resulted in incompletely abscised cells in which the two nuclei were connected by a nuclear string (n = 4). Cell binucleation occurred over a broad period, ranging from 4.6 h to 12.0 h after anaphase onset, and arose when persistent chromatin bridges in the intercellular canal induced furrow regression (Video S1). Of relevance, persistent nuclear strings connecting the two chromatin complements, presumably responsible for binucleation, were still evidenced 21.3 h after anaphase onset (Figure 6B).

Overall, these results demonstrate that p53 competent HMECs exhibiting telomere-dependent or DSBs-induced chromatin bridges engender whole-genome duplication through incomplete cytokinesis. In these cells, ultra-fine DNA bridges connecting the two chromosome complements trapped below the cleavage plane might delay abscission completion until chromatin is removed from midzone. Nevertheless, the persistence of unresolved DNA bridges can eventually induce furrow regression and generate binucleated polyploid cells, even a long time after anaphase onset.

## Discussion

The study described here clearly links progressive telomere dysfunction in human cells with epithelial cell polyploidization for the first time. Additionally, it provides a basis for the appearance of highly unstable genomes that show both structural and numerical CIN, as well as the large-scale changes in chromosome numbers that characterize epithelial cell transformation. Different mechanisms are behind the genesis of polyploid cells, but in the context of telomere dysfunction, endoreduplication cycles have been proposed as a general mechanism for the induction of tetraploidy in early stages of tumourigenesis. Mouse embryonic fibroblasts deficient in p53 and depleted of POT1a/b show an extended G2 phase and eventually bypass mitosis, which resulted in whole-genome reduplication [27]. In this genetic background, deprotection of all chromosome ends in the presence of TRF2 protein represses repair activities at uncapped telomeres and as a consequence a persistent DNA damage signal is exhibited [26]. However, age-dependent telomere erosion in humans under physiological conditions, rather than leading to an overwhelming accumulation of uncapped telomeres, might instead lead to the gradual appearance of unprotected chromosome ends that are continuously repaired by fusing with each other. In contrast to unprotected telomeres impeding the onset of anaphase [27], our findings in the HMEC model are consistent with the idea that progressive telomere shortening engenders tetraploidy, mainly through interference of end-to-end chromosome fusions with cytokinesis. Failure to conclude the cytoplasmic cell division is perceived by the accumulation of binucleated cells and it arises when trapped chromatin under the cleavage plane eventually induces furrow regression.

In humans, incomplete DNA segregation spanning the intercellular canal delays abscission completion until chromatin has been cleared from midzone [35]. Unravelling the events underpinning this new surveillance mechanism is of relevance for human cancer, since this averts furrow regression and cell tetraploidization. Several lines of evidence support the idea that the proliferation of tetraploids gradually generates subtetraploid lineages of genomically unstable cells that might fire the carcinogenic process. Almost all human cancers are genetically unstable, but the potential factor driving the acquisition of this unstable phenotype may not always be the same. A network of safeguard mechanisms and DNA repair pathways oversees the integrity of the genome. Importantly, disruption of any of these might render cells to flow through a phase of anaphase-bridge intermediates that could result in the generation of unstable tetraploids and carcinogenesis. Accordingly, defects in proteins involved in cellular processes such as sister chromatid-cohesion [36] and spindle-assembly checkpoint [37], [38], decatenation of replication intermediates [39]–[41] or even DNA-repair pathways [42], all of which have been related to cancer, culminate in the formation of chromatin bridges and binucleated tetraploid cells. As a whole, it could be concluded that failure to resolve anaphase bridges arising from the loss of telomere integrity or through different background scenarios may be a major force underlying the malignant evolution of eukaryotic cells.

Taking all things together, dysfunctional telomeres can induce tetraploidization through different mechanisms; importantly, this effect seems to be primarily dependent on the checkpoint status governed by the RB and TP53 proteins. Endoreduplication might occur when telomere damage resulting either by sheltering deficiency [27] or excessive telomere attrition [28] lasts for a considerable time period in the absence of functional p53. In such circumstances, G2/M-arrested cells are able to skip mitosis, enter into a G1-like state and are eventually licensed to reduplicate their DNA. In contrast, cytokinesis failure might prevail when progressive telomere erosion occurs in a functional p53 background. In the HMEC model, normal cultured *in vitro* cells cease proliferation due to a stress-associated senescence barrier mediated by the Rb pathway that is telomere-length independent. However, certain cell clones can overcome this barrier through the silencing of the cyclin-dependent kinase inhibitor *P16INK4A* [43]. In the absence of sufficient telomerase activity and wild-type p53, ongoing proliferation produces progressively shortened telomeres that initiate genomic instability through end-to-end chromosome fusions (reviewed by [44]). This probably corresponds to a scenario of greater physiological relevance.

Inactivation of p53/pRb pathways occurs in many tumour types in which a permissive environment for the proliferation of abnormal cells is created. Because the loss of each pathway does not occur simultaneously (reviewed by [45]), the order of p53/pRb inactivation could modulate the illicit mechanisms of whole-genome duplication. In humans, a large amount of data collected from tumour biopsies suggest that CIN is present in precancerous lesions, even before *TP53* mutations are acquired [46], [47]. In this scenario, it is therefore tempting to speculate that anaphase bridges resulting from telomere dysfunction may trigger genomic instability, in a deregulated pRb and wild-type p53 background, by fuelling highly rearranged karyotypes where structural, numerical and ploidy aberrations coexist. Eventually, the cumulative effect of centrosome-clustering induced aneuploidy [48], [49] on the preceding unstable polyploids might lead to the accumulation of highly CIN genomes with oncogenic potential; these in turn might contribute to epithelial carcinogenesis in the tissues of aged individuals.

## Materials and Methods

### Cells and culture conditions

HMECs were derived from normal breast tissue from two independent donors and were purchased from BioWhittaker (Walkersville, MD) and Cell Applications Inc. (San Diego, CA). Cells were cultured in a Human Mammary Epithelial Cell Growth Medium Kit (Cell Applications) at a temperature of 37°C in a 5% CO_2_ atmosphere. The number of accumulated PDs per passage was determined using the equation PD = PD initial+log (n° viable cells harvested/n° viable cells plated)/log2.

### Lentiviral transduction with hTERT and TRAP assay

HMECs at early PDs were transduced with viral particles containing LV.hTERT, a lentivirus construct provided by the Viral Vector Facility (CNIC; Spain), in the presence of 4 µg/ml Polybrene (Sigma-Aldrich; St. Louis, MO). After 24 h post-transduction, medium was replaced and cells were incubated at 37°C and 5% of CO_2_ atmosphere. The number of accumulated PDs after transduction was calculated according to the equation described previously. To evaluate telomerase activity, protein extracts were prepared from transduced and control cells. They were washed twice with PBS1X and lysed with 1× CHAPS Lysis Buffer (TRAPEZE Gel-Based Telomerase Detection Kit, Millipore; Billerica, MA). Protein concentration was measured with a spectrophotometer (NanoDrop 2000, Thermo Fisher Scientific; Waltham, MA). Briefly, telomere repeat amplification protocol (TRAP) assay is based on the addition of telomeric repeats to the 3′ end of a synthetic primer by telomerase, if present. In a second step, the extended products are amplified by PCR. Then, samples are mixed with 1× loading dye and resolved in a 12% non-denaturing PAGE in 0.5× TBE buffer. Gel was stained with SYBR Safe DNA gel stain (Invitrogen, Life Technologies; Carlsbad, CA) for 30–45 minutes and images were obtained using a transilluminator. A non-template control, heat-inactivated samples and cell lysates from telomerase positive cells were included as controls.

### Fluorescence *in situ* hybridization (FISH), immuno–FISH, and cell staining

#### PNA–FISH

Metaphase chromosome preparations were obtained by means of treatment with colcemid 0.02 µg/ml for 8 hours, followed by hypotonic shock and methanol/acetic fixation. Cell suspensions were dropped onto clean slides, which were stored at −20°C. Centromeres and telomeres were labeled by means of PNA-FISH techniques using a Cy3-(CCCTAA)_3_ PNA-probe for telomeres and a FITC-AAACACTCTTTTTGTAGA PNA-probe for centromeres (PE Biosystems; Foster City, CA), as previously described [50]. For evaluation of telomere dysfunction, metaphase karyotyping was performed by reverse DAPI staining, which results in a reproducible G band-like pattern that allows individual chromosomes to be identified accurately. Thereafter, pantelomeric probes allowed us to determine the chromosome arms that had signal-free telomeres (SFT). The SFT rate was obtained by dividing the number of chromosome arms without a telomere signal by the number of scored metaphases at each PD analyzed.

#### Immuno–FISH

HMECs seeded in chamber slides were grown until 70% confluence was reached. Fixation was carried out with cold methanol for 10 minutes. Cells were then permeabilized in 1×PBS-1%TritonX100 solution. The blocking step was carried out with 1×PBS-0.1%Tween20-2%Fetal Calf Serum for 1 hour at 37°C. Primary antibodies against microtubules (mouse anti alpha and beta-tubulin; Sigma-Aldrich) were diluted with blocking solution at a final concentration of 1∶500. Secondary antibody was anti-mouse Alexa-488 (1∶500; Molecular Probes, Life Technologies). Three rounds of washes with blocking solution were performed after each antibody incubation. The FISH protocol was then applied using a mixture of centromeric DNA probes specific for chromosomes 4 (CEP4; SpAqua) and 18 (CEP18; SpGreen) (Abbott Laboratories. Inc.; Downers Grove, IL), as previously described [21].

#### Texas Red-X phalloidin staining

Texas Red-X phalloidin staining (Invitrogen) was diluted with blocking solution at a final concentration of 1.5 U/ml.

Finally, all slides were dehydrated and mounted in antifade solution containing 0.125 µg/ml 4′,6-diamidino-2-phenylindole (DAPI) in antifade solution before proceeding to the microscopic analysis. Fluorescent signals were visualized under an Olympus BX60F5 epifluorescent microscope equipped with epifluorescent optics specific for each fluorochrome. Capture and analysis was carried out with the Cytovision platform (Genetix; UK).

### Flow cytometry and cell sorting

Sub-confluent HMECs were collected and fixed with ethanol 70% and kept at −20°C until analysis. Permeabilization was performed with 1×PBS-1%TritonX100 solution. The primary antibody rabbit anti-cyclin D1 (1∶100; Abcam; UK) and anti-rabbit Alexa-488 (1∶500; Molecular Probes) were applied using standard procedures that have been previously described [51]. Before acquiring the samples, they were counterstained with 0.5% Propidium Iodide (PI; 1 mg/ml) in 1×PBS-0.1%TritonX100 containing 0.2 mg/ml RNAase A DNAase-free (Sigma-Aldrich). Cell-cycle analysis was performed in a FACSCalibur. In order to prevent the cytometer recording two different cells as one event, which would result in false polyploid HMECs, doublet cells were gated out using a width-FL2/area-FL2 plot. Artificially induced binucleated cells were collected and fixed with ethanol 60%, stained with PI, and sorted with FACSCantoII (BD Biosciences; Franklin Lakes, NJ). All results were analysed with the BDFacsDiva software (BD Biosciences).

### Drug treatments

Double strand breaks were generated in HMEC-hTERT cells by the radiomimetic drug Bleocin (Calbiochem, Merck-Chemicals; Germany), a bleomycin compound, at a final concentration of 1.25 µg/ml. The drug was washed out after one hour, and cells were left recovering for 0, 6, 24, 48 and 72 hours. Cytochalasin B (Sigma-Aldrich) at a final concentration of 6 µg/ml was added to an asynchronously proliferating HMEC-hTERT culture. After 24 h, cells were collected and fixed in 60% ethanol and kept frozen until processed by FACS.

### Transfection procedures and live-cell imaging

The day prior to transfection, HMECs were plated onto a 35-mm glass bottom dish (MatTek; Ashland, MA) at a density of 7300 cells/cm^2^. Transfection procedures using a pEGFP-N1 plasmid encoding H2B-GFP sequence (BD Biosciences) were performed using Fugene HD (Roche Diagnostics S.L.; Indianapolis, IN) according to manufacturer's instructions. Live-cell imaging was performed with a Leica TCS SP5 confocal microscope. Cells were visualized with a HCX PL APO CS 40.0×1.25 OIL UV objective using the 488 nm line from an argon laser. Mitotic cells were imaged in a 3× zoom using the software Leica LAS AF Lite (Leica Microsystems; Germany) for up to 5 hours, at 3-minute intervals. Acquisition settings were established to 10% laser power through a pinhole of 5 AU, a line average of 2, and a scan speed of 400 Hz to avoid excessive cellular damage. Throughout the whole process, cells were kept at 37°C and 5%CO_2_.

To generate an immortal cell line expressing fluorescent H2B, HMEC-hTERT cells were transfected with the pEGFP-N1 plasmid encoding H2B-GFP, as described above and selected with the antibiotic Blasticidin (0.5 µg/ml; Sigma-Aldrich). Before imaging, H2B-GFP-HMEC-hTERT cells were treated with bleomycin in order to enrich the presence of anaphase bridges. Analysis was performed on an Olympus Fluoview 1000 confocal microscope, under the UPlansApo 60× objective. The Z-stacks were set with a step size of 1.5 µm. Images were acquired every 5 min. Cells were kept at 37°C and 5%CO_2_. Images were processed with ImageJ (WS Rasband, ImageJ, US National Institutes of Health, Bethesda, MD, http://imagej.nih.gov/ij/, 1997–2011), Adobe Photoshop and Adobe After Effects software.

### Statistical analysis

A Student t test was used to compare two groups of values; alternatively, a Mann-Whitney analysis was used when values did not follow a normal distribution. When 3 or more groups of data were contrasted, variance analysis for nonparametric measures (Kruscal-Wallis ANOVA) was performed. Specifically, a *post-hoc* Dunnett's test was applied to compare frequencies of anaphase bridges, binucleated cells and ploidy at the different timepoints analyzed. Correlation was calculated according to the Spearman rank correlation coefficient. A p value of less than 0.05 was considered significant.

## Supporting Information

Figure S1 DNA content exhibited by proliferating 830 HMECs. Flow cytometry analysis of DNA content of 830 HMECs stained with propidium iodide, at early and late PDs (upper panels). The percentage of cells with 4N and 8N DNA content is given. Bivariate analysis of DNA content and expression of cyclin D1 protein (lower panels), in which cyclin D1 positive cells are grouped depending on their ploidy level.
(TIF)

Figure S2 Telomere and centromere detection in non-transduced and hTERT-transduced HMECs. (A) Metaphase spread of non-transduced HMECs at PD59 hybridized with FITC-pancentromeric (green) and Cy3-pantelomeric (red) PNA probes, DNA is counterstained with DAPI. The white arrowhead indicates signal-free telomere ends (SFT) while asterisks represent end-to-end fusion events. (B) Correlation between tetraploidization events (Y axis) and the corresponding levels of SFT ends (X axis) are shown. Dots represent values for both scored parameters at different PDs, from PD25 to PD59 in donor 219-7 (blue dots) and at PD25 and PD42 in donor 830 (red dots). (C) Chromosomes of an hTERT-transduced HMEC at PD45.9 where reduced telomere instability is observed. The white arrowhead indicates signal-free telomere ends (SFT), while asterisks represent end-to-end fusion events. (D) Metaphase-spread analysis of non-transduced HMECs revealed a high presence of tetraploid cells with conventional chromosomes, and (E) only a residual presence of metaphases with duplochromosomes was observed.
(TIF)

Video S1 For the precise investigation of the process of tetraploidy development in HMECs with anaphase bridges, time-lapse imaging was performed. The video shows a HMEC-hTERT_BLEO cell expressing H2B-GFP with an over-strengthened chromatin bridge (highlighted circle) that persists unbroken and eventually leads to the formation of a binucleated cell. Total time between frames is 3 minutes; total recording time is 17.5 hours.
(MPG)
