# Supplementary figures and images for: Progressive Telomere Dysfunction Causes Cytokinesis Failure and Leads to the Accumulation of Polyploid Cells
Source: PLoS Genet. 2012 Apr 26;8(4):e1002679. doi: 10.1371/journal.pgen.1002679 (PMC3343085; doi:10.1371/journal.pgen.1002679)

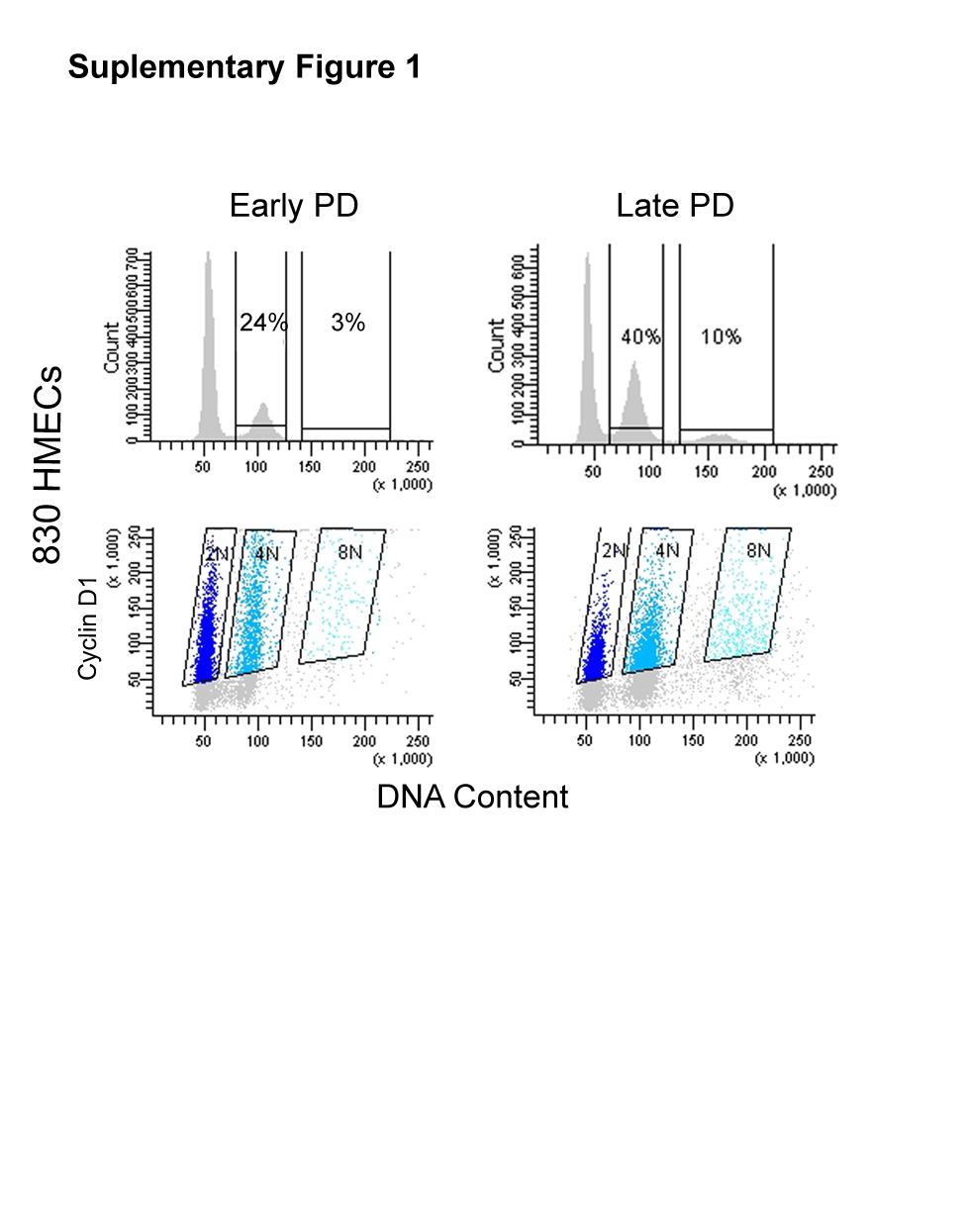

Supplement: Figure S1 — DNA content exhibited by proliferating 830 HMECs. Flow cytometry analysis of DNA content of 830 HMECs stained with propidium iodide, at early and late PDs (upper panels). The percentage of cells with 4N and 8N DNA content is given. Bivariate analysis of DNA content and expression of cyclin D1 protein (lower panels), in which cyclin D1 positive cells are grouped depending on their ploidy level. (TIF) [file pgen.1002679.s001.tif]

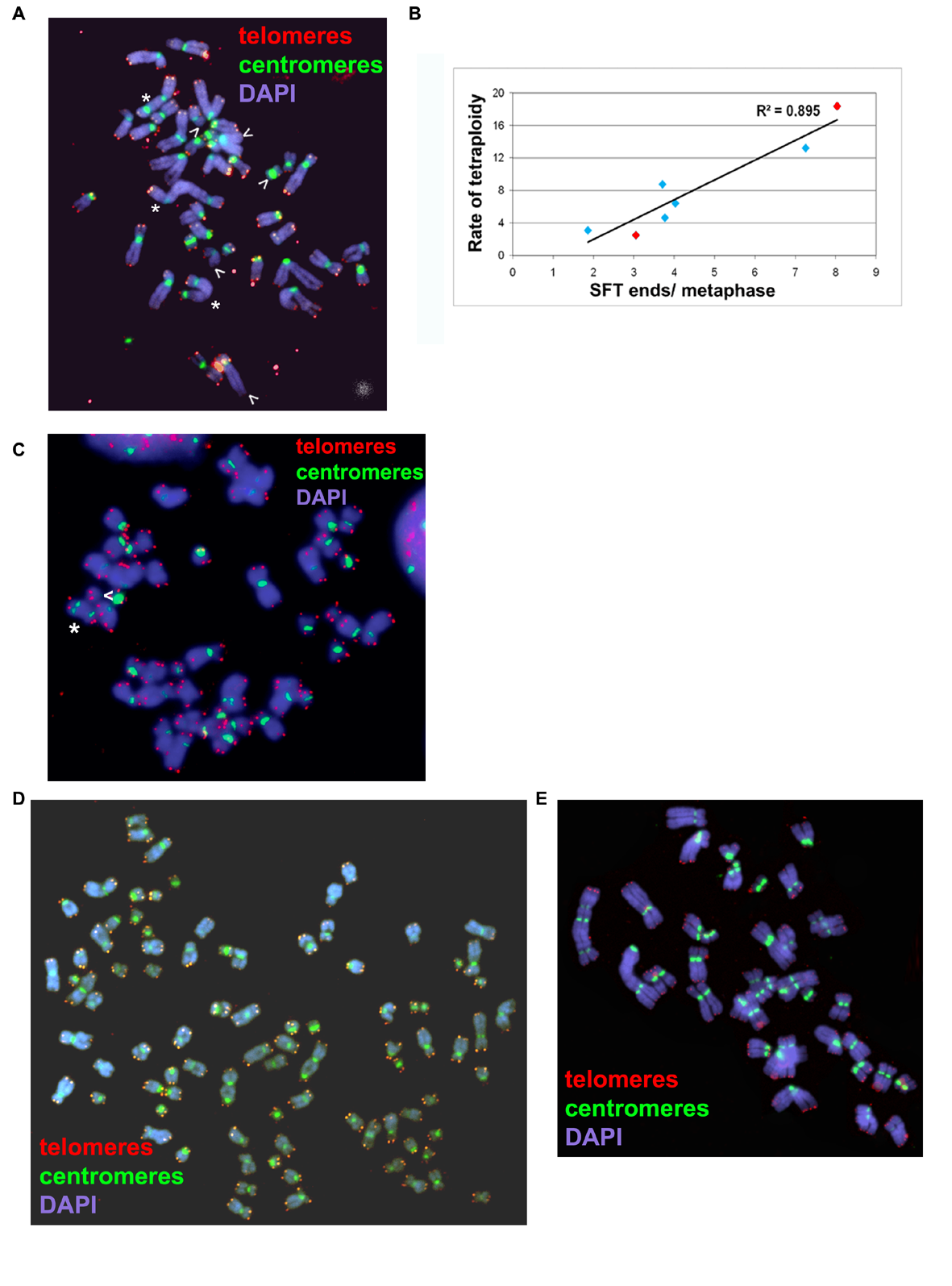

Supplement: Figure S2 — Telomere and centromere detection in non-transduced and hTERT-transduced HMECs. (A) Metaphase spread of non-transduced HMECs at PD59 hybridized with FITC-pancentromeric (green) and Cy3-pantelomeric (red) PNA probes, DNA is counterstained with DAPI. The white arrowhead indicates signal-free telomere ends (SFT) while asterisks represent end-to-end fusion events. (B) Correlation between tetraploidization events (Y axis) and the corresponding levels of SFT ends (X axis) are shown. Dots represent values for both scored parameters at different PDs, from PD25 to PD59 in donor 219-7 (blue dots) and at PD25 and PD42 in donor 830 (red dots). (C) Chromosomes of an hTERT-transduced HMEC at PD45.9 where reduced telomere instability is observed. The white arrowhead indicates signal-free telomere ends (SFT), while asterisks represent end-to-end fusion events. (D) Metaphase-spread analysis of non-transduced HMECs revealed a high presence of tetraploid cells with conventional chromosomes, and (E) only a residual presence of metaphases with duplochromosomes was observed. (TIF) [file pgen.1002679.s002.tif]
